# Reconsideration of the laminin receptor 67LR in colorectal cancer cells

**DOI:** 10.17305/bb.2024.10323

**Published:** 2024-10-01

**Authors:** Gabriel Cloutier, Jean-François Beaulieu

**Affiliations:** 1 Department of Immunology and Cell Biology, Laboratory of Intestinal Physiopathology, Faculty of Medicine and Health Sciences, Université de Sherbrooke, Sherbrooke, Canada

**Keywords:** Cell–matrix interaction, laminin receptor (LR), elastin binding protein, colorectal cancer cells

## Abstract

The 67 kDa laminin receptor (67LR) was identified as the first laminin receptor shown to be involved in the carcinogenesis of various cancers, including colorectal cancer. While the exact composition of this 67 kDa receptor remains unknown, it has been reported to be formed by the 37 kDa ribosomal protein SA (RPSA) covalently attached to another unidentified protein. The goal of this study was to clarify the molecular structure of 67LR to enhance our understanding of its role in malignancies. Using cell fractionation of colorectal cancer cells, the 67 kDa immunoreactive protein corresponding to 67LR was found in the soluble protein fraction, while some of the 37 kDa RPSA exhibited plasma membrane-like properties. Proteomic analysis of the 67 kDa fraction revealed the absence of RPSA but identified the β-galactosidase-related 67 kDa elastin-binding protein (67EBP), another laminin binding receptor which presents amino acid sequence similarities that can explain the immune cross reactivity with RPSA. The downregulation of β-galactosidase through short hairpin RNA (shRNA) led to a reduction in both 67LR and 67EBP immunoreactive proteins, confirming the misidentification of 67LR and 67EBP in colorectal cancer cells. Based on these findings, we propose to redefine the 67LR as the RPSA-containing laminin receptor (RCLR) to avoid confusion with the 67EBP.

## Introduction

Laminins, among the most abundant glycoproteins found in the intestinal basement membrane (BM) [1], play an important role in a wide variety of intestinal epithelial cell processes, namely, cell differentiation [2], proliferation [3], adhesion [4], and migration [5] through two types of cell receptors: the integrin and non-integrin receptors. These receptors include integrins, such as α7β1 [2] and α6β4 [6], and the non-integrin receptors dystroglycan [7], lutheran [8], and the 67 kDa laminin receptor (67LR) [9].

The 67LR was previously reported to be associated with cancer cells conferring aggressiveness and poor prognosis to a wide variety of cancers, such as lung [10], ovarian [11], and breast cancers [12, 13]. In colorectal cancer cells, 67LR was detected at the protein and transcript levels [14, 15] and its expression was previously correlated with tumor progression and aggressiveness [16, 17]. The receptor has also been detected in the proliferative compartment of the normal human intestinal epithelium where it regulates cell cycle and adhesion [9]. The 67LR appears to be involved in various pathological conditions, such as infection by bacteria or viruses [18], prion diseases [19], and neurodegenerative disorders [20]. Moreover, the anti-cancer effect of epigallocatechin gallate (EGCG) molecules has been shown by its action on the inhibition of ribosomal protein SA (RPSA)/67LR, suggesting that the receptor could be an interesting target for reducing tumor growth [21, 22].

The 67LR was the first identified non-integrin laminin receptor (LR) and was isolated from radiolabeled cell membranes on laminin chromatography affinity columns by several groups in the 1980s, consistent with its strong affinity for the β1 chain of laminins [23–25]. In the years following its discovery, the screening of peptide libraries with antibodies developed against the purified receptor from laminin affinity columns allowed the identification of a peptide corresponding to a sequence present on the 37 kDa RPSA [17, 26–28]. A relationship between RPSA and 67LR has been commonly accepted since two immunoreactive proteins at 37 kDa and 67 kDa on cell lysate immunoblots have been detected using antibodies developed against RPSA-sequence-derived peptides [17, 28–30]. Moreover, a pulse-chase experiment succeeded in detecting immunoreactive bands at 37 and 67 kDa with antibodies developed against the predicted sequence of RPSA suggesting that the mature high molecular weight receptor is formed from the RPSA precursor [28, 31]. This precursor hypothesis was also supported by a phylogenetic analysis of RPSA suggesting that this ribosomal protein acquired a laminin-binding sequence in evolved organisms from yeast [32].

It is noteworthy that RPSA has been found to be associated with the small ribosomal subunit of all tissues and appears to be a critical component in the initiation of translation [33]. Although RPSA is encoded by a gene corresponding to a 32.8 kDa protein, the translated protein migrates at 37 kDa on sodium dodecyl sulfate-polyacrylamide gel electrophoresis (SDS-PAGE) suggesting that post-translational modifications, such as fatty acid acylation, are involved [34, 35]. Another feature that was previously described for the 67 kDa immunoreactive protein was its resistance to reducing and denaturing conditions on SDS-PAGE suggesting that the bond between RPSA and the unknown protein forming the dimer was covalent or at least very strong [35]. Confusingly, some studies involving the 67LR focus either on the immunoreactive protein of 67 kDa [22, 34, 36–41], on the precursor form of 37 kDa which also corresponds to the ribosomal protein [20, 36, 41, 42] or both [14, 43, 44].

More recently, although the mechanism of 67LR formation from the 37 kDa precursor still remains unknown, the generally accepted theory assumes that this transition process involves post-translational modification of RPSA combined with homodimerization or heterodimerization with another yet-to-be-identified component [45, 46]. On the one hand, potential heterodimerization partners, such as galectin-3 [34, 37] or TGF-beta inhibited membrane-associated protein [47, 48], have been proposed. Although the immuno-colocalization of RPSA with galectin-3 at the cell surface during the internalization of *Neisseria meningitidis* has been recently suggested [41], this hypothesis was undermined by the expression of 67LR in N2a cells that do not express galectin-3 [49]. Despite numerous attempts to identify the dimerization partner, none have been successful in dispelling the doubt surrounding the exact identity of the mature receptor. On the other hand, the hypothesis of homodimerization has been weakened by the inability of RPSA to interact with itself in a two-yeast hybrid system [50]. Other possibilities have also been explored. For instance, the possible modification of RPSA by ubiquitin-like protein was proposed based on the detection of intermediate immunoreactive proteins of 50 kDa and 110–120 kDa with anti-RPSA antibodies [34, 51], but further studies failed to confirm this hypothesis [52]. Thus, the lingering skepticism about the relationship between the RPSA and the 67LR appears to be justified and indicates that further studies are required to elucidate the nature of this important LR.

In this study, we revisit the relationship between RPSA and the 67 kDa component of the 67LR by using different cellular fractionation methods to characterize the immunoreactive components corresponding to 67LR in colorectal cancer cells in combination with mass spectrometry analyses to define the identity of the 37 and 67 kDa immunoreactive protein-related peptides.

## Materials and methods

### Cell lines

Caco-2/15 cells were cloned from the original Caco-2 cells provided by Dr. Quaroni (Cornell University, Ithaca, NY, USA). Caco-2/15 cells were maintained in culture at sub-confluence and passed between 70% and 80% of confluence as described previously [53, 54]. HIEC-6 cells (CRL-3266) were used as a cellular model of human intestinal crypt cells [55]. The colorectal cancer cell lines SW480 (CCL-228), SW620 (CCL-227), HT29 (HTB-38), and T84 (CCL-248) were obtained from the American Type Culture Collection (Manassas, VA, USA). Caco-2/15, SW480, SW620, and HT29 cells were cultured in Dulbecco’s modified Eagle’s medium (DMEM) (Life Technologies, Burlington, ON, Canada), T84 in DMEM/F12 medium (Life Technologies), and HIEC-6 in Opti-MEM medium (Life Technologies) with 4 ng/mL EGF. All cell lines were cultured in a medium supplemented with 10% fetal bovine serum (WISENT Bioproducts, St-Bruno, QC, Canada), 2 mM Glutamax, and 10 mM HEPES (Life Technologies). Cells were routinely tested for the absence of mycoplasma contamination using the MycoSensor PCR kit (Agilent, Mississauga, ON, Canada). All cell line identities were confirmed by short-tandem repeat profiling cell authentication.

### Generation of β-galactosidase knockdown cells

Generation of stable cell lines for β-galactosidase (GLB1) knockdown cells was performed using lentiviruses prepared in HEK293T cells with the MISSION short hairpin RNA (shRNA) plasmid pLKO.1-puro (Sigma-Aldrich, Oakville, ON, Canada) as previously described [56]. The lentiviruses were prepared with shRNA plasmids containing the shRNA targeting human GLB1 sequences: 1: 5′-ATCTGTGAGGTATGTTCAGGG-3′, 2: 5′-TATATGCACCATAGTTCACAC-3′, 3: 5′-AATGCTTCCTGAGATGTAGCG-3′, 4: 5′-ATATGCACCATAGTTCACACG-3′, 5: 5′-AATCACAGGCAAAGTAGCTGC-3′. All sequences were predesigned and purchased from Sigma-Aldrich. The shRNA non-targeting was used as a control. For knockdown studies, Caco-2/15 and SW620 cells were plated at 60% confluence 24 h prior to infection with lentiviruses, infected for 48 h, and then selected with puromycin (10 µg/mL) (Qiagen, Mississauga, ON, Canada) for ten days. Stable cell lines were confirmed for knockdown by western blot and kept for further experiments.

### Protein extraction and western blot

Western blots were performed as previously described [57–59]. Briefly, cells were washed with phosphate-buffered saline (PBS), lysed using cell lysis buffer, and harvested in 1X Laemmli buffer. The lysate was then heated to 95 ^∘^C, sonicated, and centrifuged at 15,000 × *g* for 15 min. The supernatant containing the proteins was stored at –80 ^∘^C until use. The same amount of proteins was separated under denaturing and reducing conditions by 10%–15% SDS-Page and transferred to nitrocellulose membranes (BioRad). Nonspecific binding sites were blocked using 5% fat-free milk (Blotto) in PBS-Tween 20 (0.1%). The membranes were incubated overnight with the primary antibodies at 4 ^∘^C and 1 h at room temperature with the secondary antibodies coupled to HRP in the blocking solution. Positive bands were revealed using the enhanced chemiluminescence (ECL) kit (Immobilon, Millipore) and the signal was detected using either a LI-COR Biosciences/Odyssey Imager reader and Image Studio Lite 5.2 software or via autoradiographic film (GE Healthcare). Band quantification and densitometry analysis were performed using Image J software (National Institute of Health, Bethesda, MD, USA). To avoid confusion in the interpretation of the 67 kDa immunoreactive bands, membrane stripping was avoided, and the same amount of the sample was migrated separately. Relative quantification was performed based on ponceau red staining intensity or expression of β-actin. The rabbit polyclonal primary antibodies used were: anti-RPSA (Abcam, Ab90073, WB: 1/1000), anti-RPSA (Abcam, Ab246651, WB: 1/3000), anti-RPSA (Abcam, Ab99484, WB: 1/3000), anti-β-galactosidase (Abcam, Ab196858, WB: 1/1000), anti-β-galactosidase (Boster, A01829-2, WB: 1/1000), and anti-α2 integrin (Abcam, Ab133557, WB: 1/1000). A mouse anti-β-actin (Millipore, #MAB1501, WB: 1/50,000) was used for normalization in some experiments. Secondary antibodies used in immunoblotting were anti-mouse HRP-linked ECL #NA931V and anti-rabbit #NA934V (GE Healthcare, Mississauga, ON, CA, WB: 1:5000).

### Cell fractionation (protocol #1)

Cell fractionation using non-ionic detergent was performed on Caco-2/15 and SW620 cells grown to confluence. The cytosolic fraction was extracted by incubating the cell monolayer for 10 min in cytosolic extraction buffer (10 mM PIPES, 100 mM NaCl, 3 mM MgCl_2_, 5 mM EDTA, 300 mM Sucrose, 0.01% Digitonin, and 400 mM β-glycerophosphate) and then recovering the supernatant resulting from centrifugation at 480 × g for 10 min. The membrane fraction was extracted by incubating the cell monolayer for 30 min in organelles/membrane extraction buffer (10 mM PIPES, 100 mM NaCl, 3 mM MgCl_2_, 5 mM EDTA, 300 mM sucrose, Triton X-100 0.5%, and β-glycerophosphate 400 mM) and then recovering the supernatant resulting from centrifugation at 5000 × g for 10 min.

### Cell fractionation (protocol #2)

As summarized in Figure S1, 100 mm plates of Caco-2/15 cells were first rinsed twice with 1X PBS at 4 ^∘^C and then incubated for 5 min in 1.5-mL mannitol hypotonic lysis buffer (50 mM mannitol, 20 mM Tris–HCl pH 7.1, protease inhibitor [PMSF, Roche] one tablet/40 mL of buffer). Cells were transferred to a dounce type homogenizer. Approximately 50 strokes of the homogenizer were necessary to properly homogenize the cells. The effectiveness of homogenization was confirmed by visualizing the cells and cellular debris under a microscope. Following a first centrifugation at 5000 × g, to remove cellular debris and non-lysed cells, CaCl_2_ was added to the supernatant at a final concentration of 10 mM then the latter was incubated for 15 min at 4 ^∘^C and vortexed every 5 min to allow ribosome aggregation. The pellet obtained was resuspended in a volume of lysis buffer, an aliquot of the supernatant was recovered and Laemmli 4X was added to both fractions for subsequent western blot analysis. The 5000 × g supernatant from hypotonic cell lysis in mannitol buffer and ribosome precipitation was centrifuged at 20,000 × g. The pellet obtained was resuspended in a volume of lysis buffer, an aliquot of the supernatant was recovered and Laemmli 4X was added to both fractions for subsequent western blotting analysis. The remaining supernatant was recovered and ultracentrifuged at 150,000 × g for 1 h 30 min. To be centrifuged at 150,000 × g, the volume of the supernatant was increased to 14 mL with lysis buffer. The supernatant from the final ultracentrifugation was kept for analysis as a soluble fraction and the pellet containing membranes were resolubilized in lysis buffer and separated into two parts. Half of the pellet was added to a volume of lysis buffer with or without detergent (2% Triton X-100) (Sigma-Aldrich). The detergent treatment of the membrane containing pellet was carried out with constant stirring at 4 ^∘^C for 1 h 30 min followed by volume adjustment to 14 mL for ultracentrifugation at 150,000 × g for 1 h 30 min. Following the last ultracentrifugation, the final pellet was solubilized in a lysis buffer containing 4X Laemmli and the proteins contained in the supernatant were precipitated with trichloroacetic acid (Sigma-Aldrich) to a final concentration of 20% (V/V) at 4 ^∘^C. The sample was shaken vigorously and incubated for 1 h with constant stirring at 4 ^∘^C. The proteins were recovered by centrifugation at 16,000 × g and the pellet was washed three times with 100% acetone (Sigma-Aldrich). The TCA-free pellet was resuspended in Laemmli 1X buffer for western blot analysis (Figure S1).

### Cell fractionation (protocol #3)

As summarized in Figure S2, the first steps of differential centrifugation were identical to the first centrifugations at 5000, 20,000 and 150,000 × g previously described for protocol #2. However, homogenization for Caco-2/15 and SW620 cells was carried out in HEPES/sucrose lysis buffer (5 mM HEPES, 75 mM Sucrose, 20 mM Tris-HCl pH 7.1, one tablet protease inhibitor/40 mL of buffer). In addition, the pellet resulting from the ultracentrifugation at 150,000 × g was diluted in 2 mL of lysis buffer and placed on a sucrose cushion containing three concentrations: 38%, 43%, and 53%. The sucrose gradient was ultracentrifuged at 150,000 × g and recovered in five fractions. Fractions 1–4 were diluted in HEPES buffer up to 14 mL and then centrifuged at 210,000 × g for 1 h 30 min. The pellets from these four fractions were then identified as fractions F1–F4 (Figure S2).

### Mass spectrometry analyses

The fractions of interest containing the immunoreactive proteins corresponding to 67LR and RPSA were loaded onto an SDS-Page gel. Following sample migration, immunoblotting was performed in parallel with Coomassie blue gel staining as previously described. The localization of immunoreactive bands was measured with molecular weight markers and was reported on the Coomassie blue-stained gel. The extraction of the bands of interest on the gel was carried out by cutting them into several strips of approximately 1 mm^3^ and the samples were preserved in a 5% acetic acid solution.

Sample preparation and mass spectrometry experiments were performed at the Proteomic Platform (Université de Sherbrooke). The strips were first decolorized by washing in a solution of acetonitrile and 20 mM ammonium bicarbonate for 15 min. The strips were dehydrated in acetonitrile for 5 min and dried in a Speedvac for 5 min. On the other hand, the protein samples initially in lysis buffer were quantified using the BCA assay (Thermo Fisher Scientific). Following this, DTT at a final concentration of 5 mM was added to 50 µg of protein and the sample was boiled for 2 min. After a 30-min incubation at room temperature, chloroacetamide (Thermo Fisher Scientific) was added to the mixture at a final concentration of 7.5 mM and then incubated at room temperature for 20 min followed by the addition of ammonium bicarbonate (Sigma-Aldrich) to a final concentration of 20 mM.

Peptide digestion was performed overnight at 30 ^∘^C by adding 12.5 ng/mL of modified trypsin (Promega Trypsin Gold, Mass Spectrometry Grade, Promega Corporation, WI, USA) to the ammonium bicarbonate solution. Digested peptides were recovered by removing the supernatant from the digestion and cleaning the strips with a 1% formic acid solution for 20 min. The peptides thus recovered were then cleaned with ZipTip (EMD Millipore, Burlington, VT, USA).

The digested peptides were separated by high-performance liquid chromatography (HPLC) (Ultimate V3000, Thermo Fischer Scientific). Thus, 7 µL of each sample were eluted on an Acclaim PepMap100 C18 0.3 mm formic at a flow rate of 200 nL/min. The peptides from the HPLC system were then analyzed by a mass spectrometer (Orbitrap Qextractive, Thermo Fisher Scientific) with a 2.0 kV EasySpray source. A full spectrum (m/z 350–1600) was acquired at a resolution of 70,000 for 1,000,000 ions. The peptides with the greatest intensities then underwent a collision leading to their dissociation. The collision energy was adjusted to 35% and the MS/MS resolution to 17,500 for 50,000 ions with an accumulation time of 250 ms for the full scan and 60 ms for the MS/MS scan. Data were acquired by XCalibure software (Thermo Fischer Scientific) and mass spectra were analyzed using MaxQuant software. The proteins were identified by cross-checking the results obtained with the human database (Uniprot, Homo Sapiens).

### Ethical statement

The data sourced from the public database are freely accessible, therefore, this study was not required to obtain authorization from a clinical ethics committee. The study adhered to the relevant regulations of the public database.

### Statistical analysis

Each experiment was performed at least three times and representative illustrations of immunoblotting were shown. Data were expressed as mean ± standard error of the mean (SEM). The normality of the data was previously validated using the Shapiro–Wilk test at a significance level of 0.05. Subsequently, the two-tailed Student’s *t*-test for unpaired samples was used. Data presentation and statistical analyses were performed using Graph Pad Prism 8.3 (Graph Pad software; San Diego, CA, USA).

**Figure 1.**
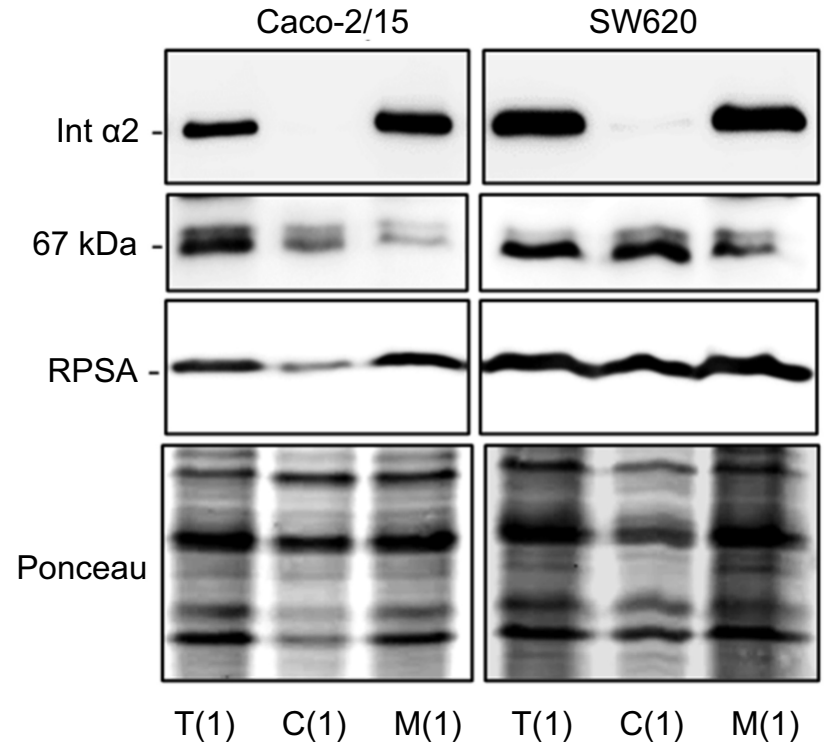
**Subcellular distribution of 67LR components following digitonin and non-ionic detergent cell fractionation.** Representative western blot showing the subcellular distribution of the 67LR immunoreactive components RPSA and the 67 kDa protein based on non-ionic detergent cell fractionation (protocol #1). Detection of the immunoreactive protein corresponding to 67LR and RPSA in total cell extract T(1), digitonin-isolated cytosolic fraction C(1), and Triton X-100-isolated membrane fraction M(1) following a sequential and differential detergent cell fractionation of colorectal cancer Caco-2/15 and SW620 cells. The detection of integrin α2 was used as a positive control for the membrane fraction and Ponceau red staining was used as a loading marker. 67LR: 67 kDa laminin receptor; RPSA: Ribosomal protein SA.

## Results

### Anti-RPSA antibodies can recognize a 67 kDa component

Western blot analyses of the 67LR were done using a panel of commercially available anti-67LR antibodies, all derived from RPSA peptides, on total protein lysates prepared from Caco-2/15 and SW620 colorectal cancer cells under reduced and denatured conditions. As shown in Figure S1, two of the antibodies, named #1 and #2, strongly detected an immunoreactive protein of 42 kDa corresponding to RPSA and another weaker band at 67 kDa that may correspond to what is considered the mature 67LR. However, antibody #3 strongly detected a 42 kDa band but failed to detect any band in the 67 kDa range (Figure S1). These results are consistent with previous observations that the 67 kDa immunoreactive protein, corresponding to the mature form of 67LR, was not sensitive to reducing or denaturing agents and conditions such as β-mercaptoethanol or SDS-Page gel electrophoresis [28, 35]. Incidentally, in the years following its discovery, the 67LR was clearly recognized as a 67 kDa protein in western blot by antibodies generated either against the purified receptor isolated from laminin affinity columns [17, 26, 30] or against RPSA-derived peptides [28, 30, 60] supporting the relationship between 67LR and RPSA. However, as shown herein, some antibodies raised against RPSA-derived peptides show no or poor detection of the 67 kDa immunoreactive protein corresponding to 67LR. For all further experiments in this study, we chose to use antibody #2 (Abcam, Ab246651), which detects RPSA as well as a 67 kDa protein, since it was still commercially available, and the sequence used as an immunogen was provided.

### Subcellular distribution of the 67LR components

Various cell fractionation methods were then used to characterize the 67LR and its immunoreactive 42 and 67 kDa components. The differential cell fractional method (referred to as protocol #1) based on the use of differential non-ionic detergent cell fractionation using digitonin to permeabilize cells and isolate the cytoplasm and Triton X-100 to solubilize and isolate the membrane fractions was first tested using the colorectal cancer cell lines Caco-2/15 and SW620. As shown in Figure 1, in colorectal cancer cells, anti-RPSA antibody detected both the 67 and the 42-kDa RPSA immunoreactive components in the digitonin-generated cytosolic fractions (lanes 2) as well as in the Triton X-100-generated membrane fractions (lanes 3). As expected, the integrin α2 subunit was only detected in the membrane fraction (Figure 1). Ponceau staining was used as a protein loading control.

**Figure 2.**
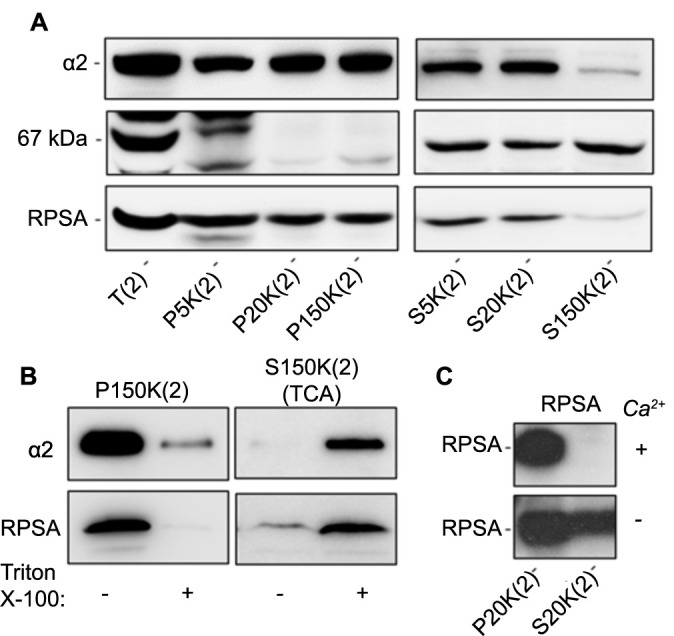
**The subcellular distribution of 67LR and RPSA using cell fractionation protocol #2.** Representative western blot analysis showing the subcellular distribution of the immunoreactive 67LR components 67 kDa protein and RPSA following differential centrifugation cell fractionation of Caco-2/15 cells in hypotonic mannitol buffer (protocol #2). (A) Detection of integrin α2 subunit and the immunoreactive 67LR component corresponding to the 67 kDa and RPSA following cell lysis in hypotonic buffer and differential centrifugation; (B) Detection of RPSA and the integrin α2 subunit in the high-speed pellets P150K(2) before and after Triton X-100 treatment (left panel) and in corresponding Triton X-100 solubilized fractions after TCA precipitation (right panel); (C) Detection of RPSA in the pellet from 20,000 × g centrifugation following cell lysis with buffer containing or not CaCl_2_; *n* ═ 3. T: Total extract; P: Pellet; S: Supernatant; (2): Lysis buffer containing mannitol (referring to protocol #2). 67LR: 67-kDa laminin receptor; RPSA: Ribosomal protein SA; TCA: Trichloroacetic acid.

To further distinguish between RPSA proteins associated with ribosomes and those involved in the formation of the mature 67LR, two cell fractionation protocols based on hypotonic cell lysis and differential centrifugation, allowing ribosome precipitation and separation of plasma membrane proteins from soluble proteins [61–63] were used (Figures S2 and S3). First, a cell lysis protocol based on mannitol hypotonic buffer followed by differential centrifugation and detergent treatment of the membrane-containing pellet (referred to as protocol #2) was performed on Caco-2/15 cells. As shown in Figure 2, the 42 kDa immunoreactive protein corresponding to RPSA was detected in the first two pellets containing the precipitated ribosomes as well as in the final pellet obtained from ultracentrifugation at 150,000 × g (P150K[2]) (Figure 2A). The detection of the integrin α2 subunit was used to confirm the specificity of the membrane and soluble fractions. Surprisingly, the 67 kDa immunoreactive protein corresponding to the mature 67LR was detected in all supernatants, including the supernatant resulting from the ultracentrifugation at 150,000 × g (S150K[2]) corresponding to the soluble protein fraction (Figure 2A). The finding of RPSA in the membrane-like containing pellet P150K(2) (Figure 2A) was further investigated using Triton X-100 solubilization as summarized (Figure S3). Triton X-100 treatment of the P150K(2) resulted in the solubilization of most of the integrin α2 subunit and RPSA (Figure 2B, left panel). TCA precipitation of the solubilized protein led to the recovery of both integrin α2 and RPSA (Figure 2B, right panel) suggesting that the RPSA detected in the P150K(2) fraction behaved similarly to the membrane protein integrin α2. As expected, the efficacy of ribosome precipitation carried out with the addition of CaCl_2_ to the hypotonic lysis buffer was confirmed as shown by the lack of significant detection of RPSA in the S20K(2) supernatant (Figure 2C). It is noteworthy that protocol #2 was ineffective for SW620 cell fractionation.

**Figure 3.**
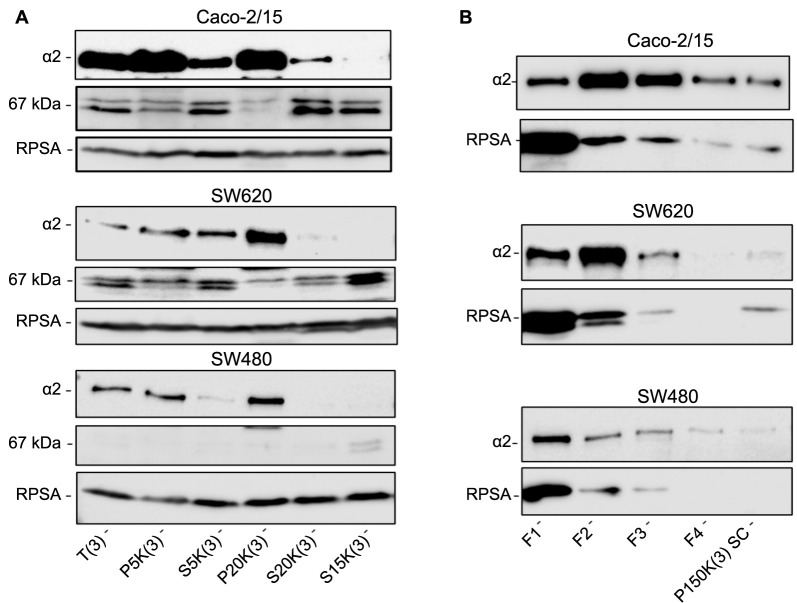
**The subcellular distribution of 67LR and RPSA using cell fractionation protocol #3.** Representative western blot analysis showing the subcellular distribution of the immunoreactive 67LR components 67 kDa protein and RPSA following differential centrifugation cell fractionation of Caco-2/15, SW620, and SW480 cells in hypotonic HEPES/sucrose buffer (protocol #3). (A) Detection of the integrin α2 subunit and the immunoreactive 67LR components 67 kDa and RPSA following cell lysis in hypotonic buffer and differential centrifugation; (B) Detection of RPSA and the integrin α2 subunit from the high-speed pellets P150K(3) after sucrose gradient. As shown in Figure S3, the F1 fraction corresponds to the overlay of the gradient while F2 to F4 are representative of the 38% to 53% sucrose gradient content while 150K(3)SC shows the pellet contents. T: Total extract; P: Pellet; S: Supernatant; F1–F4: Fraction from sucrose cushion; P150KSC: Pellet from sucrose cushion; (3): HEPES/sucrose lysis buffer (referring to samples prepared with protocol #3); 67LR: 67-kDa laminin receptor; RPSA: Ribosomal protein SA.

Considering the surprising observation that the 67 kDa component of the immunoreactive 67LR was found to be soluble, we tested a second fractionation protocol based on hypotonic lysis in the HEPES/sucrose buffer followed by separation of the membrane fraction on a sucrose cushion (referred to as protocol #3, Figure S3). For both Caco-2/15 and SW620 cells, the 67LR immunoreactive 67 kDa component was predominantly found in the supernatants including the S150K(3) supernatant containing the soluble proteins and confirmed by the absence of integrin α2 detection. However, for the SW480 cells, the detection of the 67LR immunoreactive 67 kDa was weaker for all fractions (Figure 3A). The P150K(3) fraction which contains both the integrin α2 subunit and RPSA (as shown in Figure 2A) was also further investigated on a sucrose cushion. Except for fraction 1, which corresponds to the overlay of the gradient and contains a significant proportion of RPSA, a relative proportionality of integrin α2 and RPSA was noted in the next three fractions as well as in the pellet (Figure 3B).

Overall, the results from the various cell fractionation protocols tested based on differential detergent cell fractionation and hypotonic lysis followed by differential centrifugation suggest that the 67 kDa immunoreactive 67LR component protein corresponding to 67LR remained soluble with a significant proportion being found in the digitonin-cytosolic fraction and in the 150,000 × g supernatants regardless of the lysis method used. Furthermore, these results suggest that a small proportion of the 42 kDa immunoreactive component corresponding to RPSA is detectable in the pellet of high-speed centrifugations where it displayed similar properties as the membrane protein integrin α2 subunit upon detergent treatment and in fractions of a sucrose cushion.

### Characterization of the 67LR components by mass spectrometry analyses

Mass spectrometry has been underutilized in prior attempts to characterize the 67LR and no study has managed to clearly identify the partner forming the 67-kDa mature receptor using this technique [21, 35]. This is complicated by the inability of antibodies raised against the RPSA-derived peptides to immunoprecipitate the 67 kDa form of the receptor [52]. Thus, to characterize the immunoreactive protein corresponding to 67LR components and identify the RPSA unidentified partner involved in the mature receptor, we directly analyzed the proteins isolated from the Coomassie-stained gel bands corresponding to 67LR components by mass spectrometry.

**Figure 4.**
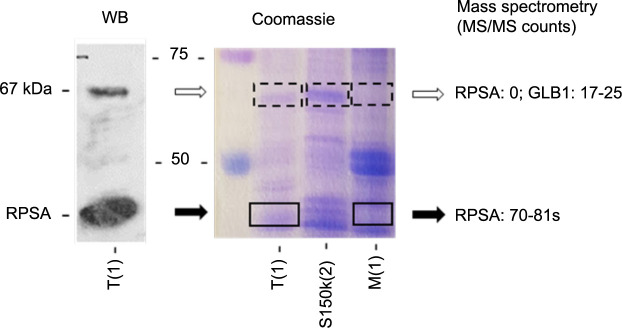
**The immunoreactive 67 kDa band associated with 67LR does not contain RPSA-related peptides.** The experimental scheme with immunoblot of total extract T(1) and Coomassie-stained SDS-page gel used to isolate the gel bands corresponding to 67LR (white arrows) and RPSA (black arrows) in the total extract, the soluble fraction from differential centrifugation cell fractionation S150K(2) and the membrane fraction from differential detergent fractionation M(1) for mass spectrometry analyses. 67LR: 67-kDa laminin receptor; RPSA: Ribosomal protein SA; WB: Western blot.

In the first set of analyses, gel bands corresponding to the immunoreactive 67 kDa and RPSA 67LR components (Figure 4, left panel) from the total extract of the protocol #1 T(1) sample (Figure 1), the S150K(2) supernatant of protocol #2 (Figure 2) and the membrane fraction M(1) of protocol #1 (Figure 1) were cut as depicted in Figure 4, (right panel) and analyzed by mass spectrometry for peptide identification. As expected, RPSA-related peptides were detected in the 42 kDa gel bands for both extract T(1) and fraction M(1) with MS/MS counts of 70 and 81, respectively (Table S1). However, no MS/MS counts were detected in the 67 kDa bands from T(1), S150K (2), or M(1) suggesting that the 67LR immunoreactive 67 kDa component is not related to RPSA. Nonetheless, several other proteins were identified from the 67 kDa bands including peptides related to GLB1 that were detected in all three fractions with MS/MS counts between 17 and 25 (Table S1). The interest of GLB1 is that its mRNA can undergo alternative splicing producing the enzymatically inactive isoform corresponding to the 67 kDa elastin binding protein (67EBP) [64] also known to have laminin-binding proprieties [65] (Figure S4). Incidentally, beside peptides common to both GLB1 and 67EPB detected, the sequence F8WF40 exclusive to 67EBP was also identified by mass spectrometry in the 67 kDa fractions strengthening the possibility that this other LR 67EBP may correspond to the hypothetical 67LR 67 kDa component. Among the other proteins identified in the 67 kDa bands, it is worth noting that no peptide related to frequently mentioned potential dimerization partners, such as galectin-3 [41], SUMO groups [52], or PEA15 [44] was detected.

**Table 1:** Solubilization of RPSA and 42 kDa membrane-associated components from the high-speed P 150K (2) pellet

**Protein name**	**Symbol**	**P150K(2) (A)**	**P150K(2) (B)**	**S150K(2) TCA (C)**
40S ribosomal protein SA	RPSA	17	2	22
Coxsackie virus and adenovirus receptor	CXADR	12	0	12
Ephrin type-B receptor 2	EPHB2	16	2	6
Flotillin-2	FLOT2	50	7	14
Sodium/potassium-transporting ATPase subunit beta-3	ATP1B3	15	3	7

MS/MS counts in the P150K (2) (A) With no Triton X-100 solubilization; (B) Remaining after Triton X-100 solubilization; (C) MS/MS counts recovered in the Triton X-100 solubilized fraction of P150K (2) after TCA precipitation. MS/MS: Tandem mass spectrometry; RPSA: Ribosomal protein SA; P150K(2): High-speed 150K pellet obtained with sample preparation according to protocol #2; TCA: Trichloroacetic acid.

**Figure 5.**
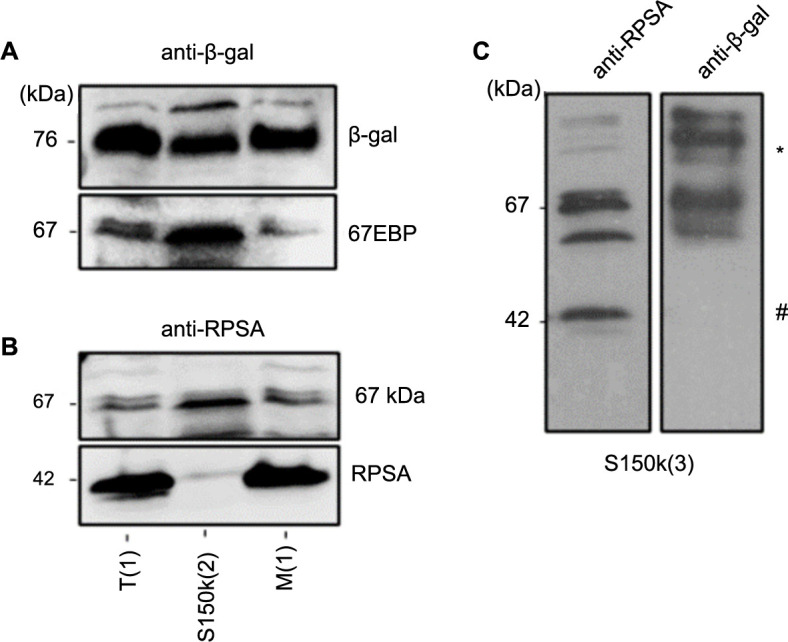
Co-detection of a 67 kDa component with both anti-β-galactosidase and anti-RPSA antibodies in intestinal cell extracts. *Enzymatic form of β-galactosidase, # RPSA. A 67 kDa immunoreactive protein corresponding to the 67EBP was found in the different 67LR positive fractions resulting from Caco-2/15 cell fractionation. (A and B) Representative western blot analyses of the total extract T(1), soluble protein fraction S150K(2) and the membrane fraction from differential detergent fractionation M(1) with anti-β-galactosidase and anti-RPSA (antibodies analyzed on separate membranes); (C) Representative western blot of soluble protein fraction S150K(3) from Caco-2/15 cells analyzed with anti-β-galactosidase and anti-RPSA antibodies showing a common set of bands at 67 kDa. 67LR: 67 kDa laminin receptor; RPSA: Ribosomal protein SA; 67EBP: 67 kDa elastin binding protein.

To ensure the cutting of the bands of the gels correspondeding to the 42 and 67 kDa regions, validation of the proteins identified in isolated gel bands analyzed via mass spectrometry was assessed by MS/MS count of the main protein-associated peptides found in each isolated gel band. As shown in Figure S5, the distribution of proteins with high sequence coverage was primarily clustered around the targeted molecular weights, confirming these proteins being present in the gel 42 and 67 kDa bands and confirming the accurate isolation of the intended molecular weight gel bands for this set of experiments.

A second set of mass spectrometry analyses was carried out on 67 and/or 42 kDa gel bands from various fractions obtained through a differential centrifugation fractionation of Caco-2/15 cells using protocol #2 (as shown in Figure 2). It is noteworthy that, as observed previously, RPSA peptides were detected in the 42 kDa bands from the total extract T(2) with 83 MS/MS counts but were absent in the 67 kDa bands (0 MS/MS counts) while GLB1-related peptides (32 MS/MS counts) were detected in the 67 kDa bands (Table S2). The identification of GLB1-related peptides (28 MS/MS counts) and no RPSA-related peptides (Table S2) in the high-speed supernatant S150K(2) confirmed that the immunoreactive band in the 67 kDa range identified with an anti-RPSA antibody (Figure 1A) was not related to 67LR. On the other hand, nothing precludes that RPSA could associate with other protein(s) to form a functional membrane receptor for laminin. In support of this possibility, the presence of RPSA-related peptides in the 42 kDa gel bands of the high-speed pellet P150K(2) was further investigated. 17 MS/MS counts for RPSA-related peptides were detected in the intact P150K(2) fraction compared to three MS/MS counts remaining after Triton X-100 treatment while 22 MS/MS counts were recovered after TCA precipitation of the solubilized fraction (Table 1 and Table S2). Interestingly, a similar pattern of distribution for galectin-3 was also identified with 11 and 5 MS/MS counts before and after Triton X-100 treatment and 8 MS/MS counts after TCA precipitation. Such a pattern appears consistent for typical membrane-associated proteins identified in the 42 kDa fraction such as CXADR, EPHB2, FLOT2, and ATP1B3 as summarized in Table 1 (see Table S2 for the complete list).

To verify that the RPSA-related peptides detected in the high-speed P150K(2) pellet are not the result of ribosome contamination, new samples were prepared according to protocol #2 corresponding to the P20K(2) and the P150K(2) pellets enriched for ribosomes (Figure 2C) and membranes (Figure 2B), respectively, and analyzed for ribosomal protein content. Few MS/MS counts of peptides related to ribosomal proteins were detected in the membrane-like containing pellet compared to the ribosome-precipitated-containing pellet (Table S3), suggesting that the RPSA-related peptides identified in the P150K(2) are not the result of ribosome contamination. Indeed, the averages for the MS/MS counts for ribosomal proteins were 37.9 for the P20K(2) and 1.3 for the P150K(2), 0.8 if RPSA as well as four other RPS proteins (RPS2, PRS3, RPS4, RPS7, and RPS8) that have been reported to have extra-ribosomal functions and localization such as at the plasma membrane [66–69] are excluded.

As above, the correct cutting of the bands of the gels for the 42 and 67 kDa regions was also validated by assessing the MS/MS count of the main protein-associated peptides found in each isolated gel band. As shown in Figure S6, the distribution of proteins with high sequence coverage was primarily clustered around the targeted molecular weights, confirming these proteins being present in the gel 42 and 67 kDa bands and confirming the accurate isolation of the intended molecular weight gel bands for this set of experiments.

Overall, these results from mass spectrometry analyses indicated that RPSA was not present in the 67 kDa immunoreactive bands corresponding to 67LR although another laminin-binding protein, 67EBP, was identified while RPSA-related peptides were found in the membrane fraction without apparent involvement of a covalent bond with another dimerization partner.

### 67EBP is detected as a 67 kDa soluble component of 67LR

To confirm the mass spectrometry finding of a 67 kDa immunoreactive protein with anti-67LR antibodies that appears to correspond to 67EBP, the fractions previously characterized by mass spectrometry were reanalyzed using an antibody against β-galactosidase. The enzymatic form of β-galactosidase and its splicing variant 67EBP (Figure S4) were detected at 76 and 67 kDa, respectively, in the total extract T(1), soluble S150K(2), and Triton X-100 membrane fraction M(1) (Figure 5A) while anti-RPSA detected a 67 kDa immunoreactive form in the three extracts (Figure 5B). Another western blot set of analyses conducted on the high-speed supernatant resulting from protocol #3 S150K(3) using anti-RPSA and anti-β-galactosidase antibodies revealed a 67 kDa immune-reactive component corresponding to both the immunoreactive 67 kDa component of the 67LR (Figure 5C, left panel) and 67EBP (Figure 5C, right panel). The immunoreactive 76 and 42 kDa components corresponding to the enzymatic β-galactosidase and RPSA were also detected with the antibodies allowing their respective recognition.

**Figure 6.**
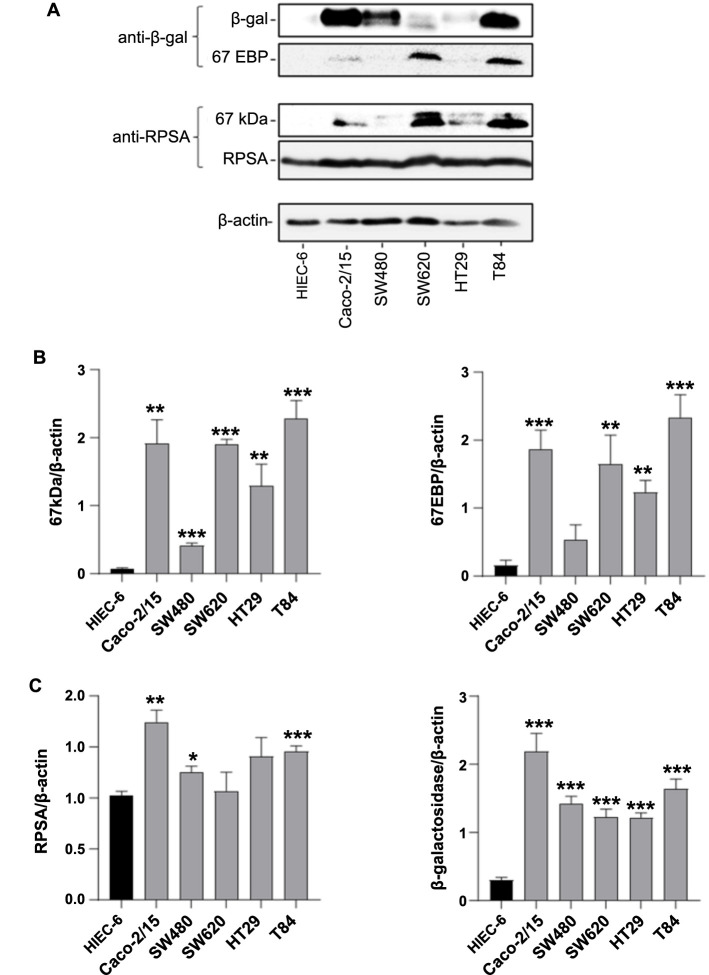
**Detection of 67LR and 67EBP in intestinal cell lines.** *P < 0.05, **P < 0.01, ***P < 0.001 vs HIEC-6 cells. Results are expressed as mean ± SEM of three separate experiments. Analysis of 67LR and 67EBP in total cell lysates of normal HIEC-6 cells and colorectal cancer cells Caco-2/15, SW480, SW620, HT29, and T84. (A) Representative western blot showing immunoreactive protein detection with anti-RPSA and anti-β-galactosidase antibodies; (B) Densitometry analysis of the 67 kDa immunoreactive proteins identified with anti-RPSA (left panel) and anti-β-galactosidase (right panel) antibodies; (C) Densitometry analysis of RPSA (left panel) and β-galactosidase (right panel). SEM: Standard error of the mean; 67LR: 67 kDa laminin receptor; RPSA: Ribosomal protein SA; 67EBP: 67 kDa elastin binding protein.

To further investigate that the 67 kDa immunoreactive protein detected with anti-RPSA antibodies and historically associated with the 67LR may corresponds to 67EBP, we analyzed the expression of immunoreactive proteins associated with the 67 kDa RPSA-immunoreactive component and 67EBP in normal HIEC-6 cells and colorectal cancer cell lines Caco-2/15, SW480, SW620, HT29, and T84. Expression of the immunoreactive 67 kDa components detected with anti-RPSA and anti-β-galactosidase antibodies were similar in all tested cell lines (Figure 6A). Thus, both 67 kDa immunoreactive proteins were strongly detected in Caco-2/15, SW620, and T84 cells compared to a weaker detection in SW480 and HT29 and no expression in HIEC-6 cells (Figure 6B). β-galactosidase was found to be strongly expressed in Caco-2/15, SW480, and T84 compared to SW620 and HT29 cells while RPSA had a relatively comparable level of expression in all cancer cell lines (Figure 6C).

### The 67 kDa immunoreactive component detected with anti-RPSA recognized 67EBP

**Figure 7.**
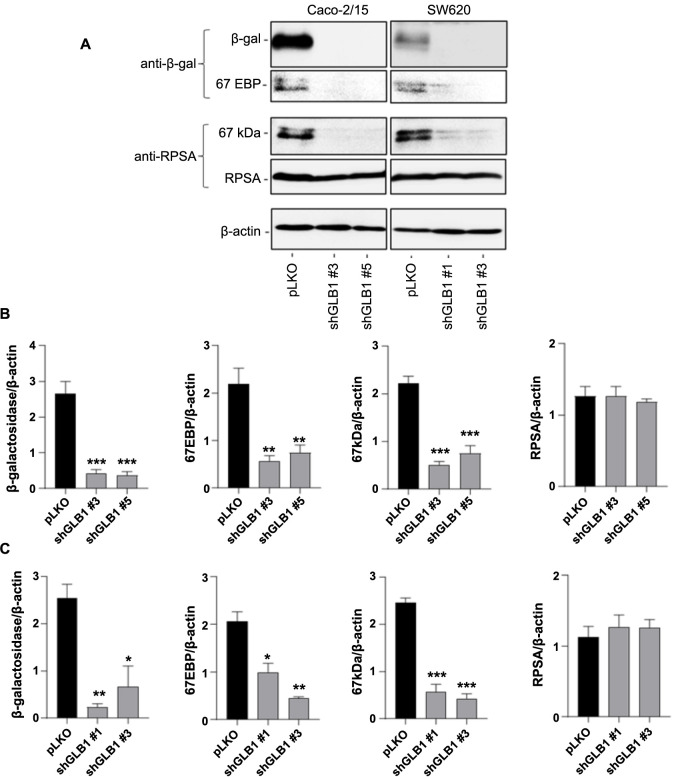
**Downregulation of β-galactosidase expression led to a reduction of the 67 kDa immunoreactive component identified with both anti-RPSA and anti-β-galactosidase antibodies.** *P < 0.05; **P < 0.01; ***P < 0.001 vs pLKO. Results are expressed as the mean ± SEM of three separate experiments. Analysis of the 67 kDa immunoreactive protein corresponding to the 67 kDa component of 67LR and 67EBP following downregulation of β-galactosidase in Caco-2/15 and SW620 colorectal cancer cells. (A) Representative western blot showing immunoreactive protein detection with anti-RPSA and anti-β-galactosidase antibodies in control and stably expressing shRNA targeting β-galactosidase cells; (B and C) Densitometry analyses of the immunoreactive proteins corresponding to β-galactosidase, 67EBP, 67 kDa component of 67LR and RPSA in Caco-2/15 (B) and SW620 cells (C). The repression of β-galactosidase led to a reduction in immunoreactive proteins associated with 67LR and 67EBP without affecting RPSA expression. SEM: Standard error of the mean; 67LR: 67-kDa laminin receptor; RPSA: Ribosomal protein SA; 67EBP: 67-kDa elastin binding protein; shRNA: Short hairpin RNA; pLKO: A replication-incompetent lentiviral vector chosen for expression of shRNAs.

The relationship between the 67 kDa component detected with the anti-RPSA antibody and 67EBP was further investigated using a down expression strategy targeting β-galactosidase in Caco-2/15 and SW620 cells to downregulate 67EBP expression. As shown in Figure 7A, total protein lysate of infected Caco-2/15 and SW620 cells with shRNA against β-galactosidase allowed a significant reduction of enzymatic β-galactosidase (76 kDa) expression as well as a significant reduction of the expression of the immunoreactive proteins corresponding to the 67EBP and 67 kDa related to RPSA compared to their respective expression in pLKO control cells (Figure 7B and 7C). As expected, the expression of the 42-kDa RPSA component was not affected by β-galactosidase repression. Results were confirmed with a second anti-β-galactosidase antibody. Overall, these results from β-galactosidase downregulation experiments indicate that the cross-reactivity of anti-RPSA antibodies with 67EBP could be the origin of confusion between these two entities.

To further document this finding, the cross-reactivity of antibodies generated against RPSA to 67EBP was verified. As shown in Table 2, analysis of the amino acid composition of the immunogen (amino acids 1–50) for the generation of antibody #2 (Abcam, Ab246651) used in this work revealed several similarities with GLB1 at the sequence level, particularly with the shared regions of GLB1 with its 67EBP isoform. Detailed information on the putative sequences responsible for the cross-reactivity of anti-RPSA antibodies with 67EBP was provided in Figure S7.

**Table 2:** Identification of putative sequences potentially responsible for the cross-reactivity of anti-RPSA antibodies raised against amino acid 1-50 (antibody #2) with 67EBP

**RPSA sequence**	67EBP sequence^*^	**Identity (%)**	**Similarity (%)**
26–30	178–182	80	100
23–28	391–395	80	100
4–8	257–261	80	100
34–38	277–282	40	100
20–27	388–398	63	75
1–8	254–261	50	75
41–48	351–358	40	63
3–10	388–395	50	63
13–22	67–76	20	60
21–56	10–24	13	53

^*^Sequences corresponding to the β-galactosidase variant 67EBP (See Figure S7 for details). RPSA: Ribosomal protein SA; 67EBP: 67-kDa elastin binding protein.

## Discussion

In this study, we suggested two important aspects that can provide a new perspective on the 67LR. Our findings in colorectal cancer cell lines pointed out that the 67 kDa immunoreactive component historically associated with the mature form of the 67LR would have been confounded with the 67EBP, a 67EBP having laminin-binding affinity through the elastin-like sequence on the β1 chain of laminin [70, 71]. Moreover, it appeared that RPSA is present at the plasma membrane as an LR without evidence of a covalent homo or heterodimerization.

As for the anti-67LR antibodies generated by using antigens obtained by laminin affinity columns [26, 30], polyclonal antibodies generated against RPSA-derived peptides were abundantly used in immunohistochemistry on various cancerous tissues for reporting an increased expression of the 67LR [10–13, 16, 72–74]. By western blot, some of these anti-RPSA antibodies were reported to detect immunoreactive proteins at 37 and 67 kDa corresponding, according to the commonly accepted theory, to the precursor (e.g., RPSA) and mature form of the 67LR [31, 45], respectively, as reported in the initial studies [28, 30, 75]. However, despite numerous studies to characterize the RPSA laminin-binding domains [46, 76, 77], none successfully characterized the entire molecular entity of 67 kDa corresponding to the mature receptor.

As mentioned above, it was noteworthy that some of the antibodies generated against the RPSA-derived peptides could recognize both 37 and 67 kDa proteins in western blot. Herein, when three of the available anti-RPSA-derived peptide antibodies were tested, all of them had a strong reaction for the detection of RPSA while two were able to detect an immunoreactive band at 67 kDa.

To further characterize the 67LR in colorectal cancer cells, we used different cell fractionation protocols that led to two unexpected findings: 1) The immunoreactive 67 kDa RPSA-related components were predominantly found under a soluble form in cell lysates and 2) Part of the cellular RPSA can be isolated in membrane protein fractions and can be released after membrane solubilization.

The finding that the immunoreactive 67 kDa component was found to be released as a soluble protein from lysates prepared with hypotonic solutions was surprising at first sight, not corresponding to the expectation for a transmembrane receptor as the 67LR [60] although the 67 kDa was found to be released from intact cells with non-ionic detergent. Analysis of these various fractions by mass spectrometry failed to identify any RPSA-related peptides in these 67 kDa gel bands, contrary to what was expected on the basis that RPSA was considered to be the precursor of the 67LR [28, 34]. Instead, a set of peptides corresponding to another laminin-binding protein of 67 kDa, the 67EBP was identified. Incidentally, the 67EBP lacked a transmembrane domain and remained associated with the plasma membrane only through its non-covalent interactions with neuraminidase-1 and cathepsin A [78, 79]. It is thus likely that hypotonic lysis contributes to 67EBP solubilization, given it was weakly retained at the plasma membrane, especially since galacto-oligosaccharides or elastin binding to 67EBP could lead to its dissociation from the membrane complex and its release into the extracellular domain [79, 80]. In intact cells, the detergent would release the complex. The detection of 67EBP in both soluble and membrane fractions would be consistent with its function which was initially described as a lysosomal protein engaged in the formation of a complex with tropoelastin allowing the biogenesis of elastin and its subsequent secretion [78, 79]. In this context, the 67EBP becomes a membrane protein during the process of elastin secretion in the extracellular domain but has also been shown to be colocalized with tropoelastin at the endoplasmic reticulum [78]. The possibility that plasma membrane-associated 67EBP could be solubilized easily was consistent with the detection of a 67 kDa immunoreactive protein in both fractions containing the membranes, during differential detergent fractionation, and in the soluble fraction during cell fractionation by differential centrifugation as observed herein. Moreover, this possibility is also supported by the fact that Caco-2/15 cells express elastin [81, 82].

Surprisingly, there have been only a few attempts to characterize the 67LR by mass spectrometry. In one of them, the 67LR isolated by laminin-affinity chromatography showed an amino acid composition similar to RPSA, thus giving rise to the possibility that the mature receptor could be a homodimer [35]. On the other hand, an interaction between EGCG and RPSA was characterized but the nature of the interaction led to the suggestion that EGCG and RPSA could be not covalently linked undermining the hypothesis of a covalent bond involving RPSA dimerization to form a mature receptor [21]. More recently, a team aiming to characterize the potential interaction between RPSA and the SUMO groups, which might be involved in the formation of an immunoreactive intermediate of 53 kDa, renounced to analyze the receptor by mass spectrometry since the 67 and 53 kDa immunoreactive proteins could not be immunoprecipitated with an antibody generated against a synthetic peptide derived from the RPSA sequence [52].

It is worth mentioning that the 67EBP was initially detected as β-galactosidase-related peptides including one identified as F8W40 which displays a sequence alignment corresponding to the alternative splicing of exon 5 associated with the emergence of the elastin binding site of 67EBP [78]. Although 67EBP is recognized as an elastin receptor, previous studies have demonstrated its capacity to interact with the elastin-like sequence found within the V domain of the β1 chain of the laminins and to be eluted from a laminin affinity column [70]. Thus, the discovery of 67LR resulting from the elution of a 67 kDa protein on a laminin affinity column and the subsequent generation of antibodies against this affinity-purified protein [23, 25] underscored the likelihood that the laminin-binding ability of 67EBP might have contributed to the confusion regarding the isolated molecular entity historically associated with 67LR.

The possibility that the identity of the 67 kDa immunoreactive protein, thought to correspond to the 67LR using RPSA/67LR antibodies, might be the 67EBP, was confirmed using a knockdown strategy using shRNA targeting β-galactosidase. The immunoreactive proteins corresponding to 67LR and 67EBP were both found to be significantly decreased in down-regulated β-galactosidase Caco-2/15 and SW620 cells while RPSA expression was not affected. The detection of an immunoreactive protein of 67 kDa with both an antibody against β-galactosidase and RPSA in the same intestinal cell lines supported the idea that 67LR might have corresponded to 67EBP.

On the other hand, it was previously suggested that the laminin-binding affinity of 67LR might be attributed to RPSA involvement within the mature receptor. Indeed, the phylogenetic analysis of homologous sequences of the RPSA gene shows, within the common ancestor of Drosophila melanogaster and vertebrates, various mutations of the region coding the amino acids 161–180 and referred to as peptide G, giving rise to the emergence of the palindromic sequence LMWWML allowing the formation of the binding site for the YIGSR peptide present on the β1 chain of laminins [32]. Furthermore, findings from X-ray crystallography and binding studies involving RPSA-derived peptides and laminin suggested that three distinct sites can interact with the β1 chain of laminins [46, 76, 83]. However, although a 67 kDa protein associated with 67LR was eluted from laminin affinity columns with a YISGR synthetic peptide [84], there are several examples in the literature for which 67LR binding to YIGSR remains unclear. Thus, in hepatocytes expressing 67LR, it seemed impossible to elute the 67 kDa receptor from affinity columns containing the YIGSR peptide in contrast to laminin affinity columns [85]. Moreover, it has previously been shown that 67LR was successfully eluted from the laminin affinity columns by peptide G while YIGSR peptides, used at higher concentrations than peptide G, failed to elute the 67LR [60] which agreed with our results suggesting that the 67 kDa immunoreactive protein did not correspond to the 67LR, but to 67EBP.

Another aspect pointed out in this study concerns RPSA as a plasma membrane LR. Indeed, treatment of the membrane fractions with two different methods, detergent solubilization and sucrose cushion separation, showed that RPSA had properties of a membrane protein, such as integrin α2. Moreover, a subsequent mass spectrometry analysis of protein extracts from the membrane-containing pellet revealed that it did not contain peptides related to exclusive ribosomal proteins in contrast to the ribosome counterpart. Incidentally, using a cell line that did not express the 67 kDa component of the 67LR, previous studies have shown the functionality of RPSA as a membrane LR by decreasing HIEC-6 cell adhesion to the immobilized YISGR synthetic peptide after reducing RPSA expression with siRNAs [9]. Moreover, RPSA detection by immunofluorescence using different antibodies directed against either the N-terminal or the C-terminal part of the protein at the membrane of permeabilized and/or non-permeabilized cells suggested the existence of a potential RPSA transmembrane domain [60]. This potential anchoring of RPSA to the plasma membrane, through a transmembrane domain, is consistent with the demonstrated detergent sensitivity solubilization of RPSA in Caco-2/15 cells. Furthermore, while a definitive demonstration of the existence of a transmembrane domain is lacking in the existing literature, an analysis of the RPSA gene sequence predicts a highly hydrophobic segment between the amino acids 86 and 105, which might potentially correspond to such a domain [28]. Alternatively, the region between amino acids 112 and 116 could also form a hydrophobic loop that could serve as an anchor and allow RPSA attachment to the plasma membrane [86]. Considering the numerous theories and hypotheses of 67LR dimerization and the well-established fact that RPSA lacks a signal peptide to regulate the trafficking of the protein, the most common explanation to explain the plasma membrane incorporation involved the second unidentified polypeptide which may possess the signal peptide or signal patch necessary for targeting the receptor [45]. Thus, although our results demonstrate that no covalent bond was involved in the formation of mature LR involving RPSA, it could not be excluded that RPSA had one or more non-covalently linked partners to form a functional membrane receptor. However, considering RPSA as a monomer receptor, the membrane association mechanism of RPSA to form a mature and functional 42 kDa LR remained questionable.

Interestingly, a new type of protein called eProts, for externalized proteins, has been identified [87]. These types of proteins were known to be located in the intracellular compartment under physiological conditions and exert an extracellular or membrane-associated localization under pathological conditions despite the absence of a signal peptide. Among the identified eProts keratin 8 [87, 88] (cytosqueletton), nucleoline [89, 90] (nucleus), ER-receptor [91] (nucleus), or GRP78 [92] (cytoplasmic) were all found either at the plasma membrane or in the extracellular domain under various pathological conditions such as cancer. The possibility that RPSA might become an externalized receptor under certain circumstances needs to be further investigated.

## Conclusion

In conclusion, we have used different cell fractionation methods to characterize the 67 kDa immunoreactive protein historically associated with the 67LR. Thus, we suggest that the cross-reactivity of some antibodies may be the cause for confusion with 67EBP, an elastin receptor also having an affinity for the β1 chains of laminin. Although our results do not definitively rule out the existence of a potential protein complex allowing the membrane incorporation of RPSA to form a potential LR, the existence of a 67 kDa dimeric receptor formed from RPSA and another covalently linked protein can be ruled out. For more clarity and to avoid confusion with the 67EBP, we propose renaming the 67LR as a RPSA-containing LR (RCLR).

## Supplemental data

Supplemental data containing figures can be found at the following link:

https://www.bjbms.org/ojs/index.php/bjbms/article/view/10323/3417

**Table S1.** **Lists of the main proteins identified in the 42 and 67 kDa bands extracted for mass spectrometry analyses from T(1), S150K(2)**, **and M(1) samples** (as shown in Figure 5) can be found at the following link:

https://www.bjbms.org/ojs/index.php/bjbms/article/view/10323/3418

**Table S2.** **Lists of the main proteins identified in the 42 and 67 kDa bands extracted for mass spectrometry analyses from T(2), P150(2)**, **and S150K(2)DT samples** (as shown in Figure 3) can be found at the following link:

https://www.bjbms.org/ojs/index.php/bjbms/article/view/10323/3419

**Table S3.** **List of the main ribosomal proteins identified in pellets corresponding to P20K(2) and P150K(2)** can be found at the following link:

https://www.bjbms.org/ojs/index.php/bjbms/article/view/10323/3420

## Data Availability

All data are contained within the article.
